# Bis(μ-*N*-benzyl-*N*-furfuryldithio­carbamato)-1:2κ^3^ *S*,*S*′:*S*′;2:1κ^3^ *S*,*S*′:*S*′-bis­[(*N*-benzyl-*N*-furfuryldithio­carbamato-κ^2^ *S*,*S*′)cadmium]

**DOI:** 10.1107/S1600536811051348

**Published:** 2011-12-03

**Authors:** Rajni Kant, Vivek K. Gupta, Kamini Kapoor, P. Valarmathi, S. Thirumaran

**Affiliations:** a X-ray Crystallography Laboratory, Post-Graduate Department of Physics & Electronics, University of Jammu, Jammu Tawi 180 006, India; b Department of Chemistry, Annamalai University, Annamalainagar 608 002, India

## Abstract

In the centrosymmetric title compound, [Cd_2_(C_13_H_12_NOS_2_)_4_], pairs of dithio­carbamate ligands exhibit different structural functions. Each of the terminal ligands is bidentately coordinated to one Cd^II^ atom and forms a planar four-membered CS_2_Cd chelate ring, whereas pairs of the tridentate bridging ligands link two neighbouring Cd^II^ atoms, forming extended eight-membered C_2_S_4_Cd_2_ tricyclic units whose geometry can be approximated by a chair conformation. The coordination polyhedron of the Cd^II^ atoms is a distorted square-pyramid. The five-membered furan ring and the benzene ring are disordered over two sets of sites with an occupancy ratio of 0.62 (8):0.38 (8).

## Related literature

For related structures, see: Ivanov *et al.* (2006 ▶); Onwudiwe & Ajibade (2010 ▶); Yin *et al.* (2004 ▶). For the solid state structural chemistry of group XII 1,1-dithiol­ates, see: Tiekink (2003 ▶). Metal dithio­carbamate complexes can act as single source precursors in the synthesis of novel metal sulfide nanomaterials, see: Ajibade *et al.* (2011 ▶); Bera *et al.* (2010 ▶); Thomas *et al.* (2011 ▶). For the effect of organic substituents on the deposition temperature and deposition mechanisms, see: Pickett & O’Brien (2001 ▶).
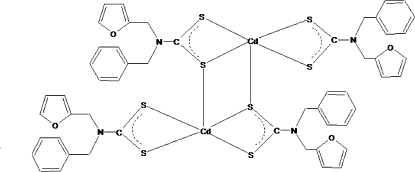


## Experimental

### 

#### Crystal data

[Cd_2_(C_13_H_12_NOS_2_)_4_]
*M* *_r_* = 1274.22
Monoclinic, 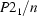

*a* = 10.7922 (3) Å
*b* = 14.7253 (6) Å
*c* = 16.9352 (6) Å
β = 97.383 (3)°
*V* = 2669.00 (16) Å^3^
*Z* = 2
Mo *K*α radiation
μ = 1.16 mm^−1^
*T* = 298 K
0.3 × 0.2 × 0.2 mm

#### Data collection

Oxford Diffraction Xcalibur Sapphire3 diffractometer
Absorption correction: multi-scan (*CrysAlis RED*; Oxford Diffraction, 2007 ▶) *T*_min_ = 0.976, *T*_max_ = 1.000
25547 measured reflections
5392 independent reflections
4546 reflections with *I* > 2σ(*I*)
*R*_int_ = 0.022

#### Refinement

*R*[*F* ^2^ > 2σ(*F* ^2^)] = 0.029
*wR*(*F* ^2^) = 0.067
*S* = 1.04
5392 reflections
337 parameters
50 restraints
H-atom parameters constrained
Δρ_max_ = 0.46 e Å^−3^
Δρ_min_ = −0.28 e Å^−3^

### 

Data collection: *CrysAlis PRO* (Oxford Diffraction, 2007 ▶); cell refinement: *CrysAlis PRO*; data reduction: *CrysAlis PRO*; program(s) used to solve structure: *SHELXS97* (Sheldrick, 2008 ▶); program(s) used to refine structure: *SHELXL97* (Sheldrick, 2008 ▶); molecular graphics: *ORTEP-3* (Farrugia, 1997 ▶); software used to prepare material for publication: *PLATON* (Spek, 2009) ▶ and *PARST* (Nardelli, 1995 ▶).

## Supplementary Material

Crystal structure: contains datablock(s) I, global. DOI: 10.1107/S1600536811051348/nc2254sup1.cif

Structure factors: contains datablock(s) I. DOI: 10.1107/S1600536811051348/nc2254Isup2.hkl

Additional supplementary materials:  crystallographic information; 3D view; checkCIF report

## Figures and Tables

**Fig. 1.**
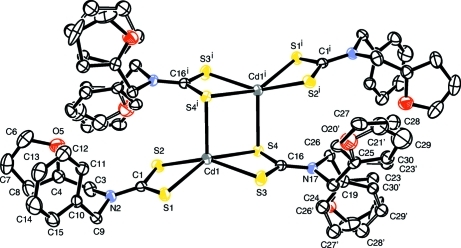
ORTEP view of the molecule with the atom-labeling scheme. The thermal ellipsoids are drawn at the 30% probability level. H atoms have been omitted for clarity. Disordered atoms are connected by full and open bonds. Symmetry codes: i = -x, -y, -z.

## supplementary crystallographic information

### Comment

The solid state structural chemistry of the group XII 1,1-dithiolates is rich and fascinating in that different structural motifs are found ranging from monomeric, dimeric, tetrameric, linear polymeric and layered structures or three-dimensional networks (Tiekink, 2003). There has been recent interest in the metal dithiocarbamate complexes, which can act as single source precursors in the synthesis of novel metal sulfide nanomaterials (Ajibade *et al.*, 2011; Bera *et al.*, 2010; Thomas *et al.*, 2011). The effect of organic substituents on the deposition temperature and deposition mechanisms have been carried out (Pickett *et al.*, 2001). This study shows that the use of dithiocarbamates as single source precursors depends on the nature of the organic substituents of the dithiocarbamate. In view of these importance we report here the first crystal structure of a dinuclear cadmium(II) dithiocarbamate complex with fufuryl substituent. The four sulfur atoms and the cadmium atom are almost coplanar. The bond angles around the cadmium atom are in the range of 67.18 (2)° to 153.03 (3). The Cd—S bond lengths range are 2.5479 (9) to 2.7952 (7) Å and are in good agreement with those reported for other Cd-dithiocarbamate complexes (Ivanov *et al.*, 2006; Onwudiwe *et al.*, 2010; Yin *et al.*, 2004). The Cd—Cd distance, which indirectly reflects the strength of the binuclear structure, is 3.751 Å.

### Experimental

*N*-Benzyl-1-(furan-2-yl)methanamine (4 mmol, 0.75 g) in ethanol was mixed with carbon disulfide (4 mmol, 0.3 ml) under ice cold condition. To the resultant yellow dithiocarbamic acid solution, Cd(CH_3_COO)_2_ 2H_2_O (2 mmol, 0.533 g) in water was added with constant stirring. The solid which precipitated was washed several times with cold water and then dried. Crystals were obtained by the slow evaporation from the solution of title compound in acetonitrile:dichloro- methane (3:1) solvent mixture (m.p. 419 K).

### Refinement

All H atoms were positioned with idealized geometry and were refined isotropic using a riding model with C—H distances of 0.93–0.97 Å and with *U*_iso_(H) = 1.2Ueq(C]. The five-membered ring (C19, O20,C21—C23 and benzene ring (C25—C30) weare disordered and were refined using a split model and site occupancy factors of 0.62 (8) and 0.38 (8) using restraints. The atoms of lower occupancy were refined only isotropic.

### Crystal data

**Table:**

[Cd_2_(C_13_H_12_NOS_2_)_4_]	*F*(000) = 1288
*M**_r_* = 1274.22	*D*_x_ = 1.586 Mg m^−^^3^
Monoclinic, *P*2_1_/*n*	Melting point: 419 K
Hall symbol: -P 2yn	Mo *K*α radiation, λ = 0.71073 Å
*a* = 10.7922 (3) Å	Cell parameters from 12062 reflections
*b* = 14.7253 (6) Å	θ = 3.2–26.3°
*c* = 16.9352 (6) Å	µ = 1.16 mm^−^^1^
β = 97.383 (3)°	*T* = 298 K
*V* = 2669.00 (16) Å^3^	Block, light brown
*Z* = 2	0.3 × 0.2 × 0.2 mm

### Data collection

**Table:**

Oxford Diffraction Xcalibur Sapphire3 diffractometer	5392 independent reflections
Radiation source: fine-focus sealed tube	4546 reflections with *I* > 2σ(*I*)
graphite	*R*_int_ = 0.022
Detector resolution: 16.1049 pixels mm^-1^	θ_max_ = 26.4°, θ_min_ = 3.2°
ω scans	*h* = −13→13
Absorption correction: multi-scan (*CrysAlis RED*; Oxford Diffraction, 2007)	*k* = −18→17
*T*_min_ = 0.976, *T*_max_ = 1.000	*l* = −21→20
25547 measured reflections	

### Refinement

**Table:**

Refinement on *F*^2^	Primary atom site location: structure-invariant direct methods
Least-squares matrix: full	Secondary atom site location: difference Fourier map
*R*[*F*^2^ > 2σ(*F*^2^)] = 0.029	Hydrogen site location: inferred from neighbouring sites
*wR*(*F*^2^) = 0.067	H-atom parameters constrained
*S* = 1.04	*w* = 1/[σ^2^(*F*_o_^2^) + (0.0273*P*)^2^ + 1.097*P*] where *P* = (*F*_o_^2^ + 2*F*_c_^2^)/3
5392 reflections	(Δ/σ)_max_ = 0.001
337 parameters	Δρ_max_ = 0.46 e Å^−^^3^
50 restraints	Δρ_min_ = −0.28 e Å^−^^3^

### Special details

**Table:**

Experimental. *CrysAlis PRO*, Oxford Diffraction Ltd., Version 1.171.34.40 (release 27–08-2010 CrysAlis171. NET) (compiled Aug 27 2010,11:50:40) Empirical absorption correction using spherical harmonics, implemented in SCALE3 ABSPACK scaling algorithm.?
Geometry. All e.s.d.'s (except the e.s.d. in the dihedral angle between two l.s. planes) are estimated using the full covariance matrix. The cell e.s.d.'s are taken into account individually in the estimation of e.s.d.'s in distances, angles and torsion angles; correlations between e.s.d.'s in cell parameters are only used when they are defined by crystal symmetry. An approximate (isotropic) treatment of cell e.s.d.'s is used for estimating e.s.d.'s involving l.s. planes.
Refinement. Refinement of *F*^2^ against ALL reflections. The weighted *R*-factor *wR* and goodness of fit *S* are based on *F*^2^, conventional *R*-factors *R* are based on *F*, with *F* set to zero for negative *F*^2^. The threshold expression of *F*^2^ > σ(*F*^2^) is used only for calculating *R*-factors(gt) *etc*. and is not relevant to the choice of reflections for refinement. *R*-factors based on *F*^2^ are statistically about twice as large as those based on *F*, and *R*- factors based on ALL data will be even larger.

### Fractional atomic coordinates and isotropic or equivalent isotropic displacement parameters (Å^2^)

**Table:**

	*x*	*y*	*z*	*U*_iso_*/*U*_eq_	Occ. (<1)
Cd1	0.670295 (16)	0.028510 (14)	0.522299 (11)	0.06295 (8)	
S1	0.88674 (6)	0.07350 (5)	0.59816 (4)	0.06729 (18)	
S2	0.81187 (6)	−0.11058 (5)	0.53613 (4)	0.06472 (17)	
S3	0.59547 (7)	0.17993 (5)	0.45861 (4)	0.0771 (2)	
S4	0.50106 (6)	0.00655 (4)	0.38421 (4)	0.05548 (15)	
C1	0.9192 (2)	−0.03999 (17)	0.59042 (13)	0.0524 (5)	
N2	1.02666 (17)	−0.07420 (14)	0.62640 (11)	0.0550 (5)	
C3	1.0579 (3)	−0.17180 (19)	0.62477 (17)	0.0728 (7)	
H31	0.9843	−0.2053	0.6024	0.087*	
H32	1.1218	−0.1807	0.5901	0.087*	
C4	1.1031 (3)	−0.20907 (18)	0.70417 (17)	0.0672 (7)	
O5	1.0175 (2)	−0.21963 (15)	0.75623 (14)	0.0909 (6)	
C6	1.0816 (5)	−0.2554 (2)	0.8242 (2)	0.1140 (13)	
H6	1.0457	−0.2696	0.8697	0.137*	
C7	1.1996 (5)	−0.2669 (3)	0.8169 (3)	0.1199 (14)	
H7	1.2611	−0.2902	0.8550	0.144*	
C8	1.2153 (3)	−0.2363 (2)	0.7378 (2)	0.0991 (11)	
H8	1.2892	−0.2356	0.7149	0.119*	
C9	1.1264 (2)	−0.01506 (18)	0.66416 (15)	0.0599 (6)	
H91	1.1190	0.0436	0.6380	0.072*	
H92	1.2062	−0.0408	0.6553	0.072*	
C10	1.1270 (2)	−0.00068 (16)	0.75214 (14)	0.0532 (5)	
C11	1.0226 (3)	−0.01013 (18)	0.79009 (16)	0.0637 (6)	
H11	0.9473	−0.0273	0.7610	0.076*	
C12	1.0282 (3)	0.0055 (2)	0.87086 (19)	0.0818 (9)	
H12	0.9576	−0.0032	0.8962	0.098*	
C13	1.1369 (4)	0.0336 (3)	0.9134 (2)	0.0993 (11)	
H13	1.1405	0.0451	0.9677	0.119*	
C14	1.2398 (4)	0.0447 (3)	0.8763 (2)	0.1085 (12)	
H14	1.3138	0.0649	0.9052	0.130*	
C15	1.2363 (3)	0.0266 (2)	0.79688 (19)	0.0826 (9)	
H15	1.3086	0.0328	0.7728	0.099*	
C16	0.4963 (2)	0.12557 (17)	0.38686 (13)	0.0542 (6)	
N17	0.42190 (17)	0.17205 (13)	0.33298 (11)	0.0534 (5)	
C18	0.3283 (2)	0.12900 (18)	0.27374 (13)	0.0570 (6)	
H18A	0.2465	0.1543	0.2783	0.068*	
H18B	0.3253	0.0644	0.2843	0.068*	
C19	0.3587 (2)	0.14361 (17)	0.19195 (14)	0.0577 (6)	
O20	0.4689 (6)	0.1278 (6)	0.1716 (3)	0.098 (2)	0.62
C21	0.4628 (9)	0.1535 (6)	0.0916 (4)	0.127 (3)	0.62
H21	0.5309	0.1576	0.0634	0.152*	0.62
C22	0.3435 (12)	0.1714 (7)	0.0617 (5)	0.146 (6)	0.62
H22	0.3134	0.1856	0.0092	0.175*	0.62
C23	0.2689 (5)	0.1636 (4)	0.1307 (3)	0.0649 (16)*	0.62
H23	0.1833	0.1706	0.1313	0.078*	0.62
C26'	0.4898 (17)	0.1293 (13)	0.1865 (9)	0.078 (5)*	0.38
H26'	0.5460	0.1139	0.2308	0.094*	0.38
C27'	0.5280 (10)	0.1396 (9)	0.1110 (7)	0.089 (4)*	0.38
H27'	0.6104	0.1285	0.1034	0.107*	0.38
C28'	0.4457 (9)	0.1654 (8)	0.0511 (7)	0.092 (4)*	0.38
H28'	0.4719	0.1681	0.0010	0.110*	0.38
C29'	0.3347 (10)	0.1865 (11)	0.0564 (8)	0.074 (4)*	0.38
H29'	0.2810	0.2061	0.0123	0.089*	0.38
C30'	0.2989 (8)	0.1800 (6)	0.1242 (5)	0.056 (2)*	0.38
H30'	0.2201	0.2037	0.1282	0.067*	0.38
C24	0.4277 (2)	0.27224 (17)	0.32614 (15)	0.0608 (6)*	
H24A	0.4322	0.2881	0.2710	0.073*	
H24B	0.5036	0.2938	0.3575	0.073*	
C25	0.3191 (2)	0.31939 (17)	0.35312 (15)	0.0596 (6)	
C26	0.3238 (6)	0.3228 (5)	0.4336 (4)	0.0671 (15)	0.62
H26	0.3874	0.2960	0.4681	0.081*	0.62
C27	0.2212 (6)	0.3718 (4)	0.4610 (3)	0.0777 (14)	0.62
H27	0.2166	0.3785	0.5151	0.093*	0.62
C28	0.1301 (7)	0.4085 (5)	0.4051 (4)	0.0790 (17)	0.62
H28	0.0653	0.4403	0.4236	0.095*	0.62
C29	0.1289 (7)	0.4014 (5)	0.3283 (5)	0.076 (2)	0.62
H29	0.0668	0.4288	0.2931	0.091*	0.62
C30	0.2188 (8)	0.3541 (6)	0.3026 (5)	0.070 (3)*	0.62
H30	0.2151	0.3433	0.2482	0.084*	0.62
O20'	0.2870 (11)	0.3447 (8)	0.4318 (7)	0.116 (4)*	0.38
C21'	0.1741 (16)	0.3940 (15)	0.4210 (11)	0.118 (8)*	0.38
H21'	0.1307	0.4173	0.4605	0.141*	0.38
C22'	0.143 (2)	0.400 (2)	0.3427 (12)	0.168 (12)*	0.38
H22'	0.0695	0.4277	0.3192	0.202*	0.38
C23'	0.2357 (12)	0.3591 (9)	0.2969 (7)	0.061 (4)*	0.38
H23'	0.2369	0.3602	0.2421	0.073*	0.38

### Atomic displacement parameters (Å^2^)

**Table:**

	*U*^11^	*U*^22^	*U*^33^	*U*^12^	*U*^13^	*U*^23^
Cd1	0.04729 (11)	0.07969 (15)	0.06005 (12)	−0.00253 (9)	−0.00010 (8)	0.01815 (9)
S1	0.0565 (4)	0.0598 (4)	0.0811 (5)	−0.0032 (3)	−0.0080 (3)	0.0110 (3)
S2	0.0555 (3)	0.0749 (4)	0.0621 (4)	−0.0052 (3)	0.0016 (3)	−0.0080 (3)
S3	0.0778 (5)	0.0687 (4)	0.0755 (5)	−0.0147 (4)	−0.0255 (4)	0.0130 (3)
S4	0.0529 (3)	0.0606 (4)	0.0525 (3)	0.0008 (3)	0.0049 (3)	0.0080 (3)
C1	0.0465 (12)	0.0664 (15)	0.0452 (12)	−0.0041 (11)	0.0098 (9)	0.0064 (10)
N2	0.0491 (10)	0.0646 (13)	0.0506 (11)	0.0028 (9)	0.0035 (8)	−0.0021 (9)
C3	0.0701 (17)	0.0738 (18)	0.0729 (18)	0.0165 (14)	0.0038 (13)	−0.0170 (14)
C4	0.0653 (16)	0.0570 (15)	0.0790 (18)	0.0081 (13)	0.0074 (14)	−0.0039 (13)
O5	0.0887 (14)	0.0842 (14)	0.1023 (17)	−0.0057 (12)	0.0218 (13)	0.0044 (13)
C6	0.169 (4)	0.074 (2)	0.100 (3)	0.003 (3)	0.020 (3)	0.025 (2)
C7	0.144 (4)	0.086 (3)	0.119 (3)	0.031 (3)	−0.024 (3)	0.020 (2)
C8	0.078 (2)	0.097 (2)	0.119 (3)	0.0280 (19)	−0.0005 (19)	0.014 (2)
C9	0.0426 (12)	0.0744 (17)	0.0618 (15)	−0.0023 (11)	0.0036 (10)	0.0039 (12)
C10	0.0508 (13)	0.0499 (13)	0.0572 (14)	0.0050 (10)	0.0001 (11)	0.0042 (10)
C11	0.0641 (16)	0.0651 (16)	0.0622 (16)	0.0047 (13)	0.0093 (12)	−0.0003 (12)
C12	0.104 (2)	0.0724 (19)	0.0727 (19)	0.0168 (17)	0.0251 (18)	0.0034 (15)
C13	0.131 (3)	0.103 (3)	0.0619 (19)	0.016 (2)	0.003 (2)	−0.0065 (17)
C14	0.102 (3)	0.139 (3)	0.077 (2)	−0.011 (2)	−0.017 (2)	−0.017 (2)
C15	0.0620 (17)	0.108 (2)	0.075 (2)	−0.0077 (16)	−0.0048 (14)	−0.0061 (17)
C16	0.0465 (12)	0.0640 (15)	0.0519 (13)	−0.0065 (11)	0.0055 (10)	0.0094 (11)
N17	0.0505 (10)	0.0560 (11)	0.0518 (11)	−0.0042 (9)	−0.0014 (8)	0.0112 (9)
C18	0.0475 (12)	0.0655 (15)	0.0556 (14)	−0.0041 (11)	−0.0032 (10)	0.0065 (11)
C19	0.0644 (15)	0.0539 (14)	0.0535 (13)	0.0033 (12)	0.0022 (11)	0.0052 (11)
O20	0.075 (3)	0.159 (5)	0.062 (3)	0.004 (3)	0.018 (3)	−0.005 (3)
C21	0.169 (10)	0.149 (8)	0.077 (5)	−0.033 (8)	0.070 (6)	−0.020 (5)
C22	0.307 (17)	0.075 (5)	0.045 (3)	0.031 (6)	−0.025 (5)	0.016 (3)
C25	0.0688 (15)	0.0532 (14)	0.0565 (15)	−0.0112 (12)	0.0068 (12)	0.0050 (11)
C26	0.069 (3)	0.068 (3)	0.070 (3)	0.003 (3)	0.026 (3)	0.000 (2)
C27	0.078 (3)	0.078 (3)	0.083 (4)	0.003 (3)	0.035 (3)	−0.012 (3)
C28	0.082 (4)	0.062 (3)	0.096 (5)	0.009 (3)	0.020 (4)	−0.002 (3)
C29	0.059 (3)	0.053 (3)	0.114 (5)	0.016 (2)	0.002 (3)	0.006 (3)

### Geometric parameters (Å, °)

**Table:**

Cd1—S2	2.5479 (7)	C18—H18B	0.9700
Cd1—S3	2.5618 (8)	C19—O20	1.300 (7)
Cd1—S1	2.6028 (7)	C19—C30'	1.353 (8)
Cd1—S4^i^	2.6355 (6)	C19—C23	1.358 (6)
Cd1—S4	2.7949 (6)	C19—C26'	1.445 (19)
S1—C1	1.716 (3)	O20—C21	1.400 (9)
S2—C1	1.730 (2)	C21—C22	1.347 (12)
S3—C16	1.711 (2)	C21—H21	0.9300
S4—C16	1.754 (3)	C22—C23	1.507 (11)
S4—Cd1^i^	2.6355 (6)	C22—H22	0.9300
C1—N2	1.337 (3)	C23—H23	0.9300
N2—C9	1.466 (3)	C26'—C27'	1.400 (15)
N2—C3	1.477 (3)	C26'—H26'	0.9300
C3—C4	1.476 (4)	C27'—C28'	1.316 (13)
C3—H31	0.9700	C27'—H27'	0.9300
C3—H32	0.9700	C28'—C29'	1.252 (13)
C4—C8	1.333 (4)	C28'—H28'	0.9300
C4—O5	1.365 (3)	C29'—C30'	1.260 (12)
O5—C6	1.369 (4)	C29'—H29'	0.9300
C6—C7	1.306 (6)	C30'—H30'	0.9300
C6—H6	0.9300	C24—C25	1.483 (4)
C7—C8	1.443 (5)	C24—H24A	0.9700
C7—H7	0.9300	C24—H24B	0.9700
C8—H8	0.9300	C25—C23'	1.356 (13)
C9—C10	1.504 (3)	C25—C26	1.359 (6)
C9—H91	0.9700	C25—C30	1.388 (8)
C9—H92	0.9700	C25—O20'	1.468 (14)
C10—C11	1.374 (4)	C26—C27	1.446 (9)
C10—C15	1.378 (4)	C26—H26	0.9300
C11—C12	1.381 (4)	C27—C28	1.384 (11)
C11—H11	0.9300	C27—H27	0.9300
C12—C13	1.359 (5)	C28—C29	1.303 (9)
C12—H12	0.9300	C28—H28	0.9300
C13—C14	1.355 (5)	C29—C30	1.314 (9)
C13—H13	0.9300	C29—H29	0.9300
C14—C15	1.366 (5)	C30—H30	0.9300
C14—H14	0.9300	O20'—C21'	1.410 (14)
C15—H15	0.9300	C21'—C22'	1.330 (16)
C16—N17	1.325 (3)	C21'—H21'	0.9300
N17—C18	1.473 (3)	C22'—C23'	1.475 (17)
N17—C24	1.482 (3)	C22'—H22'	0.9300
C18—C19	1.480 (3)	C23'—H23'	0.9300
C18—H18A	0.9700		
			
S2—Cd1—S3	153.02 (3)	O20—C19—C30'	101.5 (5)
S2—Cd1—S1	70.71 (2)	O20—C19—C23	115.1 (4)
S3—Cd1—S1	101.57 (2)	C30'—C19—C23	18.3 (4)
S2—Cd1—S4^i^	104.24 (2)	O20—C19—C26'	11.8 (8)
S3—Cd1—S4^i^	102.47 (2)	C30'—C19—C26'	111.6 (7)
S1—Cd1—S4^i^	114.07 (2)	C23—C19—C26'	126.3 (7)
S2—Cd1—S4	107.67 (2)	O20—C19—C18	122.8 (3)
S3—Cd1—S4	67.15 (2)	C30'—C19—C18	135.4 (4)
S1—Cd1—S4	153.00 (2)	C23—C19—C18	121.6 (3)
S4^i^—Cd1—S4	92.662 (19)	C26'—C19—C18	112.1 (6)
C1—S1—Cd1	83.88 (8)	C19—O20—C21	106.1 (6)
C1—S2—Cd1	85.33 (8)	C22—C21—O20	109.9 (7)
C16—S3—Cd1	91.52 (9)	C22—C21—H21	125.0
C16—S4—Cd1^i^	98.92 (8)	O20—C21—H21	125.0
C16—S4—Cd1	83.18 (8)	C21—C22—C23	105.8 (6)
Cd1^i^—S4—Cd1	87.338 (19)	C21—C22—H22	127.1
N2—C1—S1	120.37 (18)	C23—C22—H22	127.1
N2—C1—S2	119.87 (19)	C19—C23—C22	102.0 (5)
S1—C1—S2	119.76 (13)	C19—C23—H23	129.0
C1—N2—C9	121.3 (2)	C22—C23—H23	129.0
C1—N2—C3	123.0 (2)	C27'—C26'—C19	116.6 (12)
C9—N2—C3	115.4 (2)	C27'—C26'—H26'	121.7
C4—C3—N2	113.2 (2)	C19—C26'—H26'	121.7
C4—C3—H31	108.9	C28'—C27'—C26'	119.1 (12)
N2—C3—H31	108.9	C28'—C27'—H27'	120.4
C4—C3—H32	108.9	C26'—C27'—H27'	120.4
N2—C3—H32	108.9	C29'—C28'—C27'	125.0 (11)
H31—C3—H32	107.7	C29'—C28'—H28'	117.5
C8—C4—O5	110.0 (3)	C27'—C28'—H28'	117.5
C8—C4—C3	132.6 (3)	C28'—C29'—C30'	117.0 (12)
O5—C4—C3	117.4 (2)	C28'—C29'—H29'	121.5
C4—O5—C6	106.0 (3)	C30'—C29'—H29'	121.5
C7—C6—O5	111.4 (4)	C29'—C30'—C19	129.3 (9)
C7—C6—H6	124.3	C29'—C30'—H30'	115.3
O5—C6—H6	124.3	C19—C30'—H30'	115.3
C6—C7—C8	106.2 (3)	N17—C24—C25	113.5 (2)
C6—C7—H7	126.9	N17—C24—H24A	108.9
C8—C7—H7	126.9	C25—C24—H24A	108.9
C4—C8—C7	106.4 (3)	N17—C24—H24B	108.9
C4—C8—H8	126.8	C25—C24—H24B	108.9
C7—C8—H8	126.8	H24A—C24—H24B	107.7
N2—C9—C10	115.2 (2)	C23'—C25—C26	128.6 (6)
N2—C9—H91	108.5	C23'—C25—C30	9.5 (8)
C10—C9—H91	108.5	C26—C25—C30	122.0 (5)
N2—C9—H92	108.5	C23'—C25—O20'	108.3 (7)
C10—C9—H92	108.5	C26—C25—O20'	20.3 (5)
H91—C9—H92	107.5	C30—C25—O20'	102.1 (6)
C11—C10—C15	117.9 (3)	C23'—C25—C24	117.7 (6)
C11—C10—C9	123.4 (2)	C26—C25—C24	113.4 (4)
C15—C10—C9	118.7 (2)	C30—C25—C24	124.5 (4)
C10—C11—C12	120.8 (3)	O20'—C25—C24	133.4 (5)
C10—C11—H11	119.6	C25—C26—C27	114.1 (6)
C12—C11—H11	119.6	C25—C26—H26	122.9
C13—C12—C11	120.1 (3)	C27—C26—H26	122.9
C13—C12—H12	120.0	C28—C27—C26	118.8 (5)
C11—C12—H12	120.0	C28—C27—H27	120.6
C14—C13—C12	119.5 (3)	C26—C27—H27	120.6
C14—C13—H13	120.2	C29—C28—C27	124.5 (6)
C12—C13—H13	120.2	C29—C28—H28	117.7
C13—C14—C15	120.9 (3)	C27—C28—H28	117.7
C13—C14—H14	119.6	C28—C29—C30	117.4 (7)
C15—C14—H14	119.6	C28—C29—H29	121.3
C14—C15—C10	120.8 (3)	C30—C29—H29	121.3
C14—C15—H15	119.6	C29—C30—C25	122.8 (7)
C10—C15—H15	119.6	C29—C30—H30	118.6
N17—C16—S3	121.03 (19)	C25—C30—H30	118.6
N17—C16—S4	121.03 (18)	C21'—O20'—C25	108.3 (10)
S3—C16—S4	117.88 (13)	C22'—C21'—O20'	105.7 (15)
C16—N17—C18	123.3 (2)	C22'—C21'—H21'	127.2
C16—N17—C24	122.7 (2)	O20'—C21'—H21'	127.2
C18—N17—C24	114.03 (18)	C21'—C22'—C23'	113.1 (15)
N17—C18—C19	111.06 (19)	C21'—C22'—H22'	123.5
N17—C18—H18A	109.4	C23'—C22'—H22'	123.5
C19—C18—H18A	109.4	C25—C23'—C22'	104.1 (11)
N17—C18—H18B	109.4	C25—C23'—H23'	127.9
C19—C18—H18B	109.4	C22'—C23'—H23'	127.9
H18A—C18—H18B	108.0		

Symmetry codes: (i) −*x*+1, −*y*, −*z*+1.
